# Exploring Typical and Atypical Safety Climate Perceptions of Practitioners in the Repair, Maintenance, Minor Alteration and Addition (RMAA) Sector in Hong Kong

**DOI:** 10.3390/ijerph13100935

**Published:** 2016-09-22

**Authors:** Carol K.H. Hon, Yulin Liu

**Affiliations:** School of Civil Engineering and Built Environment, Queensland University of Technology, Queensland 4000, Australia; y68.liu@qut.edu.au

**Keywords:** safety climate, profile analysis via multidimensional scaling, repair, maintenance, minor alteration and addition

## Abstract

The safety of repair, maintenance, minor alteration and addition (RMAA) work is an under-explored area. This study explored the typical and atypical safety climate perceptions of practitioners in the RMAA sector in Hong Kong, based on a self-administered questionnaire survey of 662 local practitioners in the industry. Profile analysis, via multidimensional scaling of the respondents’ scores of three safety climate scales, identified one typical perception: high in management commitment to occupational health and safety (OHS) and employee involvement, low in applicability for safety rules and regulations, and low in responsibility for OHS. The respondents were clustered into typical and atypical perception groups according to their safety climate scores’ match to the typical perception. A comparison of demographics between the two groups with logistic regression found that work level and direct employer significantly affect their classification. A multivariate analysis of variance of safety performance measures between the two groups indicated that the typical group had a significantly higher level of safety compliance than the atypical group, with no significant difference in safety participation or injury. The significance of this study lies in revealing the typical safety climate perception profile pattern of RMAA works and offering a new perspective of safety climate research.

## 1. Introduction

The safety of repair, maintenance, minor alteration and addition (RMAA) has become an emergent issue that cannot be neglected [1]. It is not uncommon to find that the percentage of accidents in the RMAA sector outweighs that of its market share in the construction industry. For instance, according to Health and Safety Executive [2], remodelling, repair, extending, and building maintenance accounted for 47% of the total accidents in the construction industry of the United Kingdom in 2013/2014 whereas they only accounted for 36% of the total volume of works in the same period. Noticeably, RMAA works also have a high percentage of fatal accidents in the construction industry. For example, RMAA works accounted for 50% and 40% of the total number of fatalities in the construction industry of Hong Kong in 2013 and 2014 respectively [3]. Despite its importance, safety of RMAA works has not been given the right level of attention that it deserves [4]. 

Safety climate reflects the true priority of safety in an organization. It has been a recognized construct to predict safety performance and has been adopted in many industries as a measurement tool and leading indicator for safety [4]. For example, Occupational Safety and Health Council in Hong Kong developed a safety climate index for the adoption of the construction industry in Hong Kong [5]. Despite many research studies having been conducted, most of them focused on identifying the factors of the safety climate, establishing the relationship between the safety climate and safety outcomes, and testing the relationships of the safety climate with antecedents, moderators and mediators. There are none or only limited studies that identify individuals who have similar safety climate factor score patterns as a group, that is safety climate profile patterns, with rigorous statistical analysis. It would also be useful to know whether individuals in different profile groups have different levels of safety performance. 

This study aims to explore the heterogeneity in the safety climate. The objectives of this study are to identify the typical profile of the safety climate of RMAA works and to explore their relationships with the safety performance of RMAA works. Significance of this study lies on proffering a new perspective of analysing the safety climate. This study utilizes profile analysis via multidimensional scaling (PAMS) to identify the typical safety climate profile of RMAA works. PAMS is suitable for studies concerning types or clusters among subjects, as opposed to factors among variables in factor analysis. This is probably the first study that re-parametrises safety climate factors of the RMAA sector into profile patterns and identifies different profile groups. The identification of safety climate profile groups fills the gap of limited study on the classification of safety climate typology. The study contributes to promulgating differential safety strategies to address deficiencies observed from the typical and atypical safety climate profile groups. 

## 2. Literature Review

### 2.1. Sociotechnical Perspective of Safety

Safety is an emergent property arising from the interactions among social and technical systems [6]. Safety is the joint optimisation of an interdependency of sociotechnical systems. Joint optimisation involves interactions among system components, and between the system and its external environment [7]. Thus safety of an organization is the joint optimisation of subsystems subject to external and internal influence. While unsafe human behaviour is often seen to be the cause of accidents at the sharp end, organizational factors play an incumbent underlying role. With an increasingly complex sociotechnical system, safety management has to be holistic, addressing systemic factors at different levels. 

Carayon et al. [8] developed a conceptual model of a sociotechnical system for workplace safety. The core is work systems which are the local context in which work activities are performed. The middle layer is a social-organisational context which is comprised of social and organisational climate, culture and structure within the organization. The outermost layer is the social, economic, legal and political milieu or the external environment. Safety climate plays a role in assessing the joint optimisation of a socio-organizational level and work systems. This paper will focus on examining the interaction of a socio-organizational context and work system. More specifically, it will focus on the joint optimisation of a safety climate and workplace safety performance.

### 2.2. Safety Climate

A safety climate is the “employees” shared perceptions of their organisation’s policies, procedures and practices, as they relate to the value and importance of safety within the organisation [9]. Safety climate research started from the field of industrial and organizational psychology. Safety climate is then applied in different industrial contexts such as manufacturing. It has emerged as a useful construct to explain safety performance. A number of safety climate studies have been conducted in the construction industry (e.g., [10,11,12,13,14,15,16,17,18,19]). 

There has not been a concensus of safety climate factor structure in the construction industry. Common ones, identified from the literature, are management commitement, workers involvement and safety rules and procedures [20]. Recently, Wu et al. [21] developed a model for the core dimensions of a construction safety climate. It consisted of four core dimensions: safety priority; safety supervision, training and communication; safety rules and procedures; and safety involvement. Wu et al. [21] agrued that while the safety climate factor structure can vary in different contexts, the core dimensions, which are the essense of the safety climate, should not be changed.

As for the RMAA sector, Hon et al. [20] examined the safety climate factors of RMAA works. Three factors were determined, namely factor 1, management commitment to OHS and employee involvement; factor 2, applicability of safety rules and work practices; and factor 3, responsibility for health and safety. While many studies examined the safety climate factors, there is limited information on the profile distribution of these safety climate factors and the classification of safety climates. 

Regarding the classification of safety climates, Lingard et al. [16] developed a typology for the classification of group safety climates. Safety climate can be assessed according to the level and the strength. Lingard et al.’s study was probably the first to reveal different types of safety climates in the construction industry. Safety climate strength is the degree of consensus concerning climate perceptions within members of a group and can range from weak to strong. The level of safety climate refers to the relative priority placed upon safety within a workgroup as perceived by members of that group. Based on the climate level and climate strength, a typology consisting of four types of safety climate were determined, they are:

- An indifferent safety climate (weak strength and low level);
- An obstructive safety climate (strong strength and low level);
- A contradictory climate (weak strength and high level);
- A strongly supportive climate (strong strength and high level).

An indifferent safety climate represents a low level of commitment to safety and low level of consensus to the relative priority placed on safety. Obstructive safety climate represents a strong consensus that safety is not so important to the organization. A contradictory climate represents a mixed message of safety importance. Workers may pay lip service. A strong, supportive safety climate represents a consistently high priority to safety without compromise on different circumstances. In Lingard et al. [16], amongst the 307 respondents, which represented 40 workgroups in road construction and maintenance organisations, hospital construction projects and national steel reinforcement manufacturing organizations in Australia, the predominant types of workgroup safety climate were “strongly supportive” (*n* = 14, 35%) or “indifferent” (*n* = 14, 35%) whereas the less predominant types were obstructing (*n* = 6, 15%) contradictory (*n* = 6, 15%). Lingard et al. [16] revealed that there were predominant types and less predominant types of safety climate exisiting in the construction industry. 

The study of Zhou et al. [18] in China identified four safety climate factors, namely (1) safety attitude; (2) safety supervision, safety training and workmates’ support; (3) management commitment; and (4) safety regulations. The mean scores of safety climate factors would shed some light on the distribution of the levels of safety climate factors. Mean scores, in a 5-point Likert scale, in descending order were (1) safety attitude (4.13); (2) safety supervision, safety training and workmates’ support (3.91); (3) management commitment (3.86); and (4) safety regulations (3.46). While the safety climate factor mean scores are useful indicators, the typical profile pattern of safety climate factors in Chinese construction companies is yet to be explored.

## 3. Research Methods

### 3.1. Questionnaire

A questionnaire survey was conducted with RMAA contractors in Hong Kong. There were three parts in the questionnaire. Part A was questions for personal particulars. Part B adopted the Safety Climate Index (SCI) survey developed by the Occupational Safety and Health Council (OSHC) of Hong Kong. The SCI was selected because it was readily available in both English and Chinese and was designed for the adoption of the construction industry in Hong Kong. Part C was questions regarding safety performance: injuries, safety participation, and safety compliance. 

RMAA safety climate: The RMAA safety climate has been investigated in Hon et al. [20]. Hon et al’s [20] study shows that the safety climate of RMAA works in Hong Kong consists of three factors encapsulating 22 questions (Appendix Table A1) of the Safety Climate Index (SCI) survey of the Occupational Safety and Health Council (OSHC) of Hong Kong [5]. Three RMAA safety climate factors were: management commitment to OHS and employee involvement, applicability of safety rules and work practices, and responsibility for health and safety. These questions were evaluated by the respondents in a five-point Likert scale, with “1” being “strongly disagree” and “5” being “strongly agree”. These three factors will be adopted for further analysis in this paper. 

Self-reported near misses and injuries: Four questions were utilized to capture the near misses and occupational injuries of the respondents in the last 12 months with a 5-point ordinal scale (0 = Never; 1 = 1 time; 2 = 2–3 times; 3 = 4–5 times; 4 = Over 5 times). The questions were: (1) How many times have you been exposed to a near miss incident of any kind at work? (2) How many times have you suffered from an injury of any kind at work, but did not require absence from work? (3) How many times have you suffered from an injury, which required absence from work not exceeding 3 consecutive days? (4) How many times have you suffered from injuries, which required absence from work exceeding 3 consecutive days?

Safety participation: With reference to Neal and Griffin [22], two questions were modified to measure safety participation of the respondents with a 5-point Likert scale. The two questions were: (1) How frequently do you put in extra effort to improve the safety of the workplace (e.g., reminding coworkers about safety procedures at work)? (2) How frequently do you voluntarily carry out tasks or activities that help to improve workplace safety (e.g., attending safety meetings, receiving safety training)?

Safety compliance: With reference to Mohamed [12], two questions were set to measure, in terms of time (0% to 100%), the degree of safety compliance to all safety procedures by the respondents and their co-workers respectively. The two questions were: (1) Do you follow all of the safety procedures for the jobs that you perform? (2) Do your coworkers follow all of the safety procedures for the jobs that they perform?

### 3.2. Participants and Procedure

The questionnaire survey was conducted between April and August 2009. The pilot questionnaire was vetted by a group of advisory group members of the research project. These advisory group members mainly provided industry support for carrying out the research. A sampling framework consisting of clients, property management companies, RMAA contractors and subcontractors was designed. The finalized questionnaires were dispatched through industrial links of the advisory group members. With their facilitation, a number of private property management companies, maintenance sections of quasi-government developers and their subcontractors, RMAA sections of general contractors, small RMAA contractors, building services contractors and trade unions in Hong Kong, participated in this study. A total of 844 questionnaires were sent out and 814 completed questionnaires were returned from managers, supervisors and workers. The response rate was 96.3%. Such a high response rate was attributed to the advisory group members’ support of data collection in their companies and their industry networks. After deleting some outliers and incomplete questionnaires, a total of 662 questionnaires were valid for analysis in this paper. Regarding the working level of the respondents, 397 were frontline workers, 131 were supervisors, 129 were managers, and five did not indicate their working level. Regarding the type of company that the respondents worked for, 70 respondents worked for the clients, 248 worked for the main contractors, 297 worked for the subcontractors, and 32 did not provide any information.

### 3.3. Analysis 

Multidimensional scaling profile analysis was employed to identify the typical profile(s) of safety climate factors among the survey respondents. As a person-oriented approach, multidimensional scaling profiling explicitly targets the identification of unobserved heterogeneity in a population on the basis of the observed data [23]. Multidimensional scaling enables representation of typical (normative) profiles within the population and simultaneously demonstrates how individuals differ with the individual regression’s model fit (R^2^) and the profile match index to each profile (i.e., regression coefficient). This approach to profile analysis is exploratory and is most suited to situations where normative profiles are derived from data rather than specified by a particular theory [24]. Kim et al. [25] compared multidimensional scaling profile analysis with cluster analysis: one major limitation of cluster analysis is that the clusters describe individual differences in overall cluster level, rather than individual differences in profile patterns.

The multidimensional scaling profile analysis model for a set of psychological variables in the data has the following general form:

$$y_{i\left(v\right)}=c_{i}+\underset{k}{\sum }w_{ik}x_{k\left(v\right)}+e_{i\left(v\right)}$$

where $y_{i\left(v\right)}$ is an observed score of individual *i* for the *v*th variable. $c_{i}$ is a level parameter estimate for individual *i*, which corresponds to his/her average score across all variables. $w_{ik}$ is a profile match index for individual *i* on dimension *k*. $x_{k\left(v\right)}$ is the latent profile parameter estimate (a.k.a., scale value) for variable *v* along dimension *k*, and $e_{i\left(v\right)}$ is an error term for individual *i* for variable *v*. One of the major goals of multidimensional scaling profile analysis is to determine the number of prototypical profiles expressed in scale values.

Proximity scaling (PROXSCAL) procedure in SPSS23 is employed for the multidimensional scaling because PROXSCAL treats small and large distances equally to find an optimal solution, thus it is now generally preferred. Dissimilarity is measured as Euclidean distance between variables or safety climate scales in this study. The multidimensional scaling procedure is seeking an ordinal model with simplex as the initial solution. The number of dimensions will be determined based on model fit indices and the cross-validation result using randomised sample halves. The resulting latent profile scale values will be used to predict individual profiles with a standard linear regression.

For identifying atypical individuals, an individual’s fit to the model (R^2^) is assessed. Each individual’s model fit indicates the proportion of variation in his/her observed profile that can be accounted for by prototypical profile(s) derived from a multidimensional profile model. The higher the R^2^ value, the better the person-model fit. This approach makes it possible to identify individuals who develop in an idiographic manner, indicating that the overall model, typical of most people, does not apply to a particular person [23]. Instead of using 0.40 as the cut-off value of an individual model fit (R^2^) to classify typical and atypical individuals [23], this study employs the two-step cluster algorithm in SPSS23 to explore the natural grouping among the survey respondents. The distance between two clusters is measured as the decrease in the log-likelihood when they are combined into a single cluster. The optimal number of clusters is selected based on Schwarz Bayesian information criterion. Cluster analysis is an exploratory technique (i.e., there are no significance tests in the clustering solution). This method was selected due to the very new nature of this field of research; future research may use different methods to confirm or refute the findings presented here.

As a follow-up analysis of the clustering analysis, logistic regression is to be performed to compare between clusters in terms of their members’ demographics; a multivariate analysis of variance (MANOVA) is to be used to compare average safety performance indicators including self-report injuries, safety participation, and safety compliance between the clusters.

## 4. Results

Cross-validation, using randomised sample halves, confirmed that one dimension is the optimal solution of the multidimensional scaling model in this study. Kruskal’s Stress (Stress-1) is the most commonly adopted measure of the (mis)fit of a multidimensional scaling model. Stress-1 being zero indicates a perfect solution and larger values indicate a poorer fit. At the bottom line, a sensible multidimensional scaling solution’s Stress should be substantially less than the value expected for random data [26]. Kruskal and Wish [27] proposed that Stress-1 less than 0.05 is excellent and Stress-1 larger than 0.20 is poor. The one-dimensional multidimensional scaling solution of this study’s data showed an excellent model fit with a Stress-1 value less than 0.01. Table 1 presents the scale values of safety climate factors in a multidimensional scaling model. As suggested by the profile scale values, the dominant profile pattern of the respondent is high in Factor 1 but low in Factors 2 and 3. This indicates that RMAA works practitioners have high perception on management commitment to OHS and employee involvement; but low perception on applicability of safety rules and work practices; and responsibility for health and safety. 

Further, each respondent’s individual safety climate profile is regressed on the three scale values.

**Figure 1:**
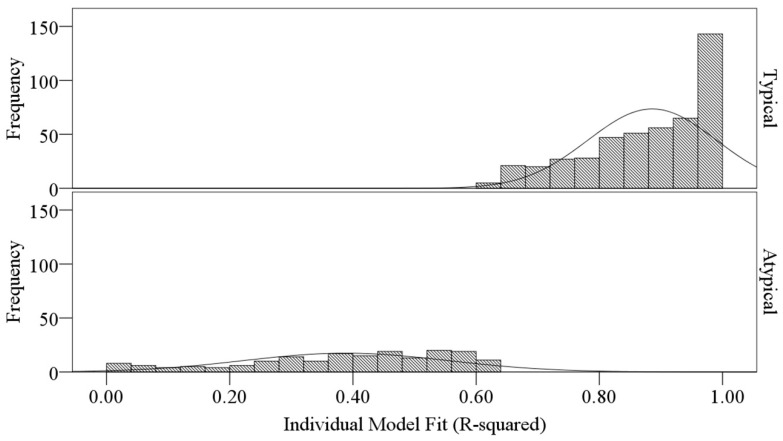
Histograms of individual model fit (R^2^) for typical and atypical groups.

Instead of using 0.4 as an arbitrary cut-off value [23], two-step cluster analysis was employed to find natural clusters among the respondents based on their individual model fit (R^2^). It resulted in two distinct clusters with an average Silhouette coefficient (that theoretically ranges from −1.0 to 1.0) of 0.8, which indicates a very good cluster solution, namely the individuals within a cluster are similar to one (or cohesive) while the clusters themselves are quite different (or separated) [28]. One cluster (*n* = 481) is 2.66 times larger than the other (*n* = 181). The two clusters are respectively named typical and atypical groups because the individual model fit (R^2^) values in the typical group are higher than those in the atypical group. Summary statistics in Table 2 and histograms in Figure 1 compare the individual model fit (R^2^) between the two clusters.

To compare the individuals’ characteristics between the typical and atypical groups, a binary logistic regression was performed to predict the atypical group membership based on a range of demographics of the respondents (*n* = 621 with listwise exclusion of missing values). The regression model showed omnibus statistical significance (−2*LL* = 687.427, *χ*^2^(13) = 37.636, *p* < 0.001) and modest goodness of fit (Nagelkerke R^2^ = 0.085, Hosmer and Lemeshow test *χ*^2^(8) = 2.781, *p* = 0.947) with an overall rate of correct classification of 73%. Table 3 presents the coefficient estimates of the logistic regression and compares the two groups in terms of the explanatory variables. Significant variables include working level and director employer. Respondents working as supervisors and managers have significantly higher chances of being classified as the atypical group as compared with those working as frontline workers. Respondents working for the main contractors have significantly lower chances of being classified as atypical as compared with those working for clients. The atypical group has a higher percentage of supervisors (29.2%) and managers (25.6%) than that of the typical group, which are 17.7% respectively. Besides, the atypical group has more than doubled the percentage of respondents working for the sub-contractors (18.5%) than the typical group (7.9%).

MANOVA was performed to test effects of typical/atypical profile grouping on safety performance indicated by self-report injuries, safety participation, and safety compliance. A Box’s test confirmed the assumption of equivalent observed covariance matrices of the three dependent variables across the two groups (M = 5.827, *F*(6, 738,185.7) = 0.965, *p* = 0.447). With this assumption being met, MANOVA results indicated that there were significant multivariate between-subjects effects for group (V = 0.029, *F*(3658) = 6.621, *p* < 0.001). Further, between-subjects ANOVA was conducted as a univariate test for each outcome variable. All the dependent variables satisfied the assumption of homogeneous variances between two groups, with Levene’s test *p* values ranging from 0.133 to 0.921. Univariate tests found that the grouping effect is statistically significant only on safety compliance (*F*(1660) = 19.134, *p* < 0.001), but not significant on self-reported injuries (*F*(1660) = 1.812, *p* < 0.179) or safety participation (*F*(1660) = 1.661, *p* < 0.198) at the 5% level. This indicates that the typical group has a significantly higher level of safety compliance as compared with that of the atypical group. 

## 5. Discussion

This research has revealed the typical profile pattern of the safety climate of RMAA practitioners in Hong Kong. The pattern is high regarding *Management commitment to OHS and employee involvement*; but low regarding *Applicability of safety rules and work practices*; and *Responsibility for health and safety*. This result shows that the RMAA practitioners, in general, have a positive perception towards the management commitment of RMAA companies to uphold safety and the safety involvement of employees. However, the respondents have a negative perception of the applicability of safety rules and work practices and their own responsibility for health and safety. In contrast to the study of Zhou et al. [18] that management commitment scored the second lowest among all the safety climate factors, management commitment in the RMAA sector scored the highest in the profile analysis and was perceived to have achieved a higher level than the other factors. This shows that RMAA contracting companies that participated in this research have been doing well in safety management. Due to convenient sampling, it is noteworthy that the companies that were willing to participate in this research may represent those companies that are more committed to safety and have more established safety management systems. 

It is not surprising to find that applicability of safety rules and work practices has a low score in profile analysis. The existing safety rules and work practices were mainly designed for new construction sites. Some of the rules or regulations are not applicable or hard to adopt in the RMAA sector. For example, it is dangerous to conduct live electrical work; however, in reality, unless there is good coordination with property management, it is hard to switch off the power to carry out repair and maintenance [20]. Tasks of RMAA works are often minute and can be completed in a short time. The efforts required to invest in observing the safety rules and regulations may well be greater than completing the tasks [1]. This refrains practitioners from observing tedious safety procedures and undermines their usefulness. However, it is surprising to find that the respondents perceived the responsibility for health and safety to be low. This finding may imply that the RMAA practitioners have a passive attitude for health and safety or they have a low perceived locus of control over safety and health issues [29]. Based on the low profile score, the RMAA practitioners’ sense of responsibility for health and safety seems to be inadequate.

What would be the implications of these profile analysis results? The typical safety climate profile of the RMAA sector reveals deficiencies in safety management of the RMAA companies that require improvements. It seems that more efforts should be focused on reviewing the safety rules and regulations governing the RMAA sector and building up the safety ownership of the practitioners of the RMAA sector. RMAA works often involve minute tasks that can be completed in a short time. Following stipulated safety measures may take many times longer than completing the tasks. This makes the rules and regulations impractical to follow. In view of the rising importance of RMAA works, it is time to review and design a set of safety rules and best practice for the RMAA sector to adopt. It has to be recognised that the RMAA sector has unique features that are different from the new construction sector. Instead of using the same set of safety management system, rules and regulations, there is a need to reconsider the best approach to tackle safety hazards of the RMAA sector separately. The RMAA contracting companies should consider streamlining the safety rules and procedures which allow more flexibility to suit the nature of their jobs. 

The practitioners of the RMAA sector need to have a strong safety ownership for self-monitoring because most RMAA works do not have the same level of safety supervision as new construction works due to a small scale and short project duration. They should proactively take precautionary measures to protect themselves as well as their workmates. Safety culture is cultivated from the top by the management and from the bottom by the employees of the company. The RMAA practitioners would need to change their safety attitude from passive to proactive. To promote a positive safety culture of the RMAA practitioners, concerted efforts among the contractors, property owners, relevant government departments and Occupational Safety and Health Council, are needed. 

Noticeably, the atypical group has significantly lower safety compliance than the atypical group. The atypical group consists of a higher percentage of respondents directly employed by subcontractors. There are many possible reasons leading to subcontractors’ lower level of safety compliance. Small subcontractors may not have proper safety management practice and the resources for safety to enforce compliance with safety rules. Some may have a cowboy attitude towards safety. Some may be over-confident with their experience and thus neglect the importance of safety compliance. Whatever the reason may be, it is suggested that the government or the Occupational Safety and Health Council should put more effort into uplifting the safety awareness and knowledge of RMAA practitioners at the subcontractors’ level [30].

## 6. Conclusions, Limitations and Implications 

To conclude, this paper has revealed the typical safety climate profile pattern of the RMAA sector practitioners in Hong Kong. Management commitment to OHS and employee’ involvement has the highest profile score, whereas applicability of safety rules and regulations and responsibility for health and safety have relatively lower profile scores. The profile analysis findings indicate that more effort has to be put into reviewing and designing proper safety rules and regulations for the RMAA sector and cultivating the safety ownership of the RMAA practitioners so that they have a stronger sense of responsibility for health and safety. The atypical group has a significantly lower level of safety compliance than the typical group. It is noticed that the atypical group has a higher proportion of respondents working for subcontractors. Subcontractors’ safety awareness and knowledge has to be uplifted. 

This study is limited by convenient sampling. The sampling framework was determined by the industry networks of the advisory group members of the research project. Hence, participants of this study were mainly employed by RMAA contracting companies that were well-known to the advisory group members or the RMAA section of the main contractors. Those one-man band RMAA contracting companies were not included in this study and they may have a different safety climate profile from those relatively established players in this study. Although this study was conducted in Hong Kong, the findings would be applicable to other places with an expanding RMAA sector having similar safety challenges. 

This study contributes to providing another perspective to investigate the safety climate, and reveals deficiencies for further improvement. The findings would be useful for the government, RMAA contracting companies, and safety managers to promulgate relevant safety precautionary measures for the RMAA sector. Perhaps the most important implication of the safety climate profile findings to the industry is that efforts in improving the safety climate of the RMAA sector need to shift from management commitment at the company level to safety ownership at the indivudal level. 

## Figures and Tables

**Table 1:** Scale values of safety climate factors in multidimensional scaling model (*n* = 662).

Safety Climate Factors	Profile Scale Value
Factor 1: Management commitment to occupational safety and health (OSH) and employee involvement	0.816
Factor 2: Applicability of safety rules and work practices	−0.400
Factor 3: Responsibility for health and safety	−0.417

**Table 2:** Individual model fit (R^2^) of typical and atypical groups.

Cluster	Mean	*n*	*SD*	Median	Kurtosis	Skewness	Min.	Max.
Typical	0.89	481	0.10	0.91	−0.45	−0.76	0.627	1.000
Atypical	0.39	181	0.17	0.42	−0.38	−0.68	0.001	0.625
Total	0.75	662	0.25	0.84	0.20	−1.07	0.001	1.000

**Table 3:** Coefficient estimates of the logistic regression to predict the atypical group (*n* = 621).

Explanatory Variable	Category	Typical Group (%, *n* = 453)	Atypical Group (%, *n* = 168)	B	*p*	95% CI for Odds Ratio	
						Lower	Upper
Working level	Frontline worker ^b^	64.6	45.2	-	-	-	-
	Supervisor	17.7	29.2	0.798	0.003	1.301	3.796
	Manager	17.7	25.6	0.977	0.002	1.417	4.980
Age group in 10 years ^a^	20 or less	1.5	1.8	−0.232	0.100	0.602	1.046
	21 to 30	21.2	20.2				
	31 to 40	29.8	36.6				
	41 to 50	33.1	31.0				
	51 to 60	13.7	10.7				
	61 or more	0.7	0.0				
Marital status	Single ^b^	68.4	67.3	-	-	-	-
	Married	31.6	32.7	−0.124	0.632	0.533	1.465
Highest level of education	Primary or below ^b^	60.3	59.5	-	-	-	-
	Secondary or Diploma	24.7	18.5	0.224	0.447	0.702	2.228
	Degree or higher	15.0	22.0	−0.197	0.499	0.463	1.455
Direct employer	Client ^b^	50.6	50.5	-	-	-	-
	Main contractor	41.5	31.0	−0.535	0.018	0.375	0.913
	Subcontractor	7.9	18.5	0.434	0.183	0.814	2.927
Length of service with current company	5 years or less ^b^	59.6	60.7	-	-	-	-
	6 to 10 years	18.3	10.1	−0.464	0.130	0.345	1.145
	11 years or more	22.1	29.2	0.015	0.957	0.597	1.726
Working experience in construction industry	5 years or less ^b^	19.9	22.6	-	-	-	-
	6 to 15 years	45.9	41.1	−0.315	0.258	0.423	1.259
	16 years or more	34.2	36.3	−0.198	0.576	0.410	1.642
Safety training qualification ^a^	No green card	0.9	0.6	0.013	0.937	0.733	1.400
	Green card	70.4	69.0				
	Green card + trade specific/silver card/others	24.3	26.8				
	Green card + any two (trade specific/silver card/others)	4.0	3.6				
	Green card + trade specific + silver card + others	0.4	0.0				
Constant	-	-	-	−0.214	0.725	-	-

Notes: ^a^ included as a continuous scale in the logistic regression; ^b^ reference category the categorical explanatory variable.

## Appendix

**Table A1:** 22 statements measuring RMAA safety climate.

***Factor 1 Management commitment to OHS and employee involvement***
The company really cares about the health and safety of the people who work here
There are good communications here between management and workers about health and safety issues
The company encourages suggestions on how to improve health and safety
I am clear about what my responsibilities are for health and safety
I think management here does enough to follow up recommendations from safety inspection and accident investigation reports
All the people who work in my team are fully committed to health and safety
There is good preparedness for emergency here
Accidents which happened here are always reported
Most of the job-specific safety trainings I received are effective
I fully understand the health and safety risks associated
Safety inspection here is helpful to improve the health and safety of workers
Staff are praised for working safely
***Factor 2 Applicability of safety rules and work practices***
Some jobs here are difficult to do safely
Not all the health and safety rules or procedures are strictly followed here
Some of the workforces pay little attention to health and safety
Some health and safety rules or procedures are difficult to follow
Supervisors sometimes turn a blind eye to people who are not observing the health and safety procedures
Sometimes it is necessary to take risks to get the job done
***Factor 3 Responsibility for health and safety***
People are just unlucky when they suffer from an accident
Accident investigations are mainly used to identify who should be blamed
Work health and safety is not my concern
Little is done to prevent accidents until someone gets injured

## References

[1] Hon C.K.H., Chan A.P.C., Wong F.K.W. (2010). An analysis for the causes of accidents of repair, maintenance, alteration and addition works in Hong Kong. Saf. Sci..

[2] Health and Safety in Construction in Great Britain, 2014/2015. http://www.hse.gov.uk/statistics/industry/construction/construction.pdf.

[3] Work Safety Performance of Repair, Maintenance, Alteration and Addition Works. http://www.legco.gov.hk/yr14-15/english/panels/mp/papers/mp20150317cb2-1044-3-e.pdf.

[4] Chan A.P.C., Hon C.K.H. (2016). Safety of Repair, Maintenance, Minor Alteration, and Addition (RMAA) Works: A New Focus of Construction Safety.

[5] Occupational Safety and Health Council (OSHC) (2008). Construction Industry Safety Climate Index Software.

[6] Leveson N.G. (2011). Applying systems thinking to analyse and learn from events. Saf. Sci..

[7] Hendrick H.W., Kleiner B.M. (2001). Macroergonomics: An Introduction to Work System.

[8] Carayon P., Hancock P., Leveson N., Noy I., Sznelwar L., van Hootegem G. (2015). Advancing an sociotechnical systems approach to workplace safety—Developing the conceptual framework. Ergonomics.

[9] Zohar D., Quick J.C., Tetrick L.E. (2011). Safety climate: Conceptual and measurement issues. Handbook of Occupational Health Psychology.

[10] Dedobbeleer N., Béland F. (1991). A safety climate measure for construction sites. J. Saf. Res..

[11] Glendon A.I., Litherland D.K. (2001). Safety climate factors, group differences and safety behavior in road construction. Saf. Sci..

[12] Mohamed S. (2002). Safety climate in construction site environments. J. Constr. Eng. Manag..

[13] Fang D., Chen Y., Wong L. (2006). Safety climate in construction industry: A case study in Hong Kong. J. Constr. Eng. Manag..

[14] Choudhry R.M., Fang D., Lingard H. (2009). Measuring safety climate of a construction company. J. Constr. Eng. Manag..

[15] Lingard H., Cooke T., Blismas N. (2009). Group-level safety climate in the Australian construction industry: Within-group homogeneity and between-group differences in road construction and maintenance. Constr. Manag. Econ..

[16] Lingard H., Cooke T., Blismas N. (2010). Properties of group safety climate in construction: The development and evaluation of a typology. Constr. Manag. Econ..

[17] Lingard H., Cooke T., Blismas N. (2011). Co-workers’ response to occupational health and safety: An overlooked dimension of group-level safety climate in the construction industry. Eng. Constr. Archit. Manag..

[18] Zhou Q., Fang D., Mohamed S. (2011). Safety climate improvement: Case study in a Chinese Construction Company. J. Constr. Eng. Manag..

[19] Zhang R.P., Lingard H., Nevin S. (2015). Development and validation of a multilevel safety climate measurement tool in the construction industry. Constr. Manag. Econ..

[20] Hon C.K.H., Chan A.P.C., Yam M.C.H. (2013). Determining safety climate factors in the repair, maintenance, minor alteration, and addition sector of Hong Kong. J. Constr. Eng. Manag..

[21] Wu C., Song X., Wang T., Fang D. (2015). Core dimensions of the construction safety climate for a standardized safety climate measurement. J. Constr. Eng. Manag..

[22] Neal A., Griffin M.A. (2006). A study of the lagged relationships among safety climate, safety motivation, safety behaviour, and accidents at the individual and group levels. J. Appl. Psychol..

[23] Dong Y., Ding C. (2012). Adolescent risk behaviors: Studying typical and atypical individuals via multidimensional scaling profile analysis. J. Adolesc..

[24] Ding C.S. (2006). Multidimensional scaling modelling approach to latent profile analysis in psychological research. Int. J. Psychol..

[25] Kim S.K., Frisby C.L., Davison M.L. (2004). Estimating cognitive profiles using profile analysis via multidimensional scaling (PAMS). Multivar. Behav. Res..

[26] Borg I., Groenen P.J.F., Mair P. (2013). Applied Multidimensional Scaling.

[27] Kruskal J.B., Wish M. (1978). Multidimensional Scaling.

[28] Norusis M.J. (2012). IBM SPSS Statistics 19 Statistical Procedures Companion.

[29] Hinze J. (2006). Construction Safety.

[30] Hon C.K.H., Chan A.P.C., Chan D.W.M. (2011). Strategies for improving safety performance of repair, maintenance, minor alteration and addition (RMAA) works. Facilities.

